# Continuous development in macromolecular crystallography with CCP4

**DOI:** 10.1107/S205979832300445X

**Published:** 2023-05-30

**Authors:** Martín Martinez-Ripoll, Armando Albert

**Affiliations:** a Instituto de Química Física Blas Cabrera, Consejo Superior de Investigaciones Científicas, Calle Serrano 119, Madrid, 28006, Spain

**Keywords:** Collaborative Computational Project No. 4, CCP4, X-ray crystallography software, macromolecular crystallography

## Abstract

The evolution of the Collaborative Computational Project No. 4 (CCP4) has been described in a new article by Agirre *et al.* [(2023). *Acta Cryst.* D**79**, 449–461] that should provide the definitive reference for the *CCP*4 suite of programs.

People engaged in X-ray crystallography since the early 1970s have been spectators and beneficiaries of the contributions of many colleagues who dedicated a significant part of their professional careers to the development of computational methodology for X-ray crystallography. Indeed, from the earliest standalone programs for processing experimental measurements extracted from Weissenberg diagrams, to the handling of the resolution and refinement programs and the earliest graphic representation programs, crystallographers will always be grateful to this community effort. But over the years, small-molecule crystallographers benefited from the great advances made in the development of integrated systems, such as the *XRAY*76 System, which for the first time offered a full collection of tools for full resolution of small-molecule structures from single-crystal X-ray diffraction data.

The field of macromolecular crystallography also underwent a development equivalent to that of small molecules, although somewhat later. The establishment of the Collaborative Computational Project No. 4 (known as CCP4) in 1979 represented a spectacular qualitative advance in the field. CCP4 is a public resource for producing and supporting a world-leading, integrated suite of programs that allows researchers to determine and analyze macromolecular structures by X-ray crystallography. Indeed, the CCP4 project has evolved in line with structural biology advancements, becoming an essential resource for researchers and scientists worldwide (Fig. 1 ▸).

Nowadays, the CCP4 initiative is not merely a collection of programs, it includes a graphical interface that guides less experienced users through the resolution pathway to the structural analysis and provides traceability of jobs. In addition, CCP4 supports various educational projects around the globe. These initiatives extend beyond self-promotion and include an open mailing list that anyone can join to share conference and workshop announcements, questions and discussions, that sometimes turn into debates, and information about job offers.

The article by Agirre *et al.* in this issue (Agirre *et al.*, 2023 ▸), provides a thorough review of the *CCP*4 suite of programs. It constitutes a long-awaited initiative intended to review the current state of the CCP4 project and future developments. In addition, it offers an up-to-date literature citation for the suite, which has had multiple references in the past, and for each of the individual programs. The review aligns with the idea that the complexity of macromolecular crystallography should be accessible to both non-expert and advanced users and should offer computing tools regardless of the difficulty of the project. The article also provides a critical analysis of the challenges and opportunities that the field of macromolecular crystallography faces now and in the future.

## Figures and Tables

**Figure 1:**
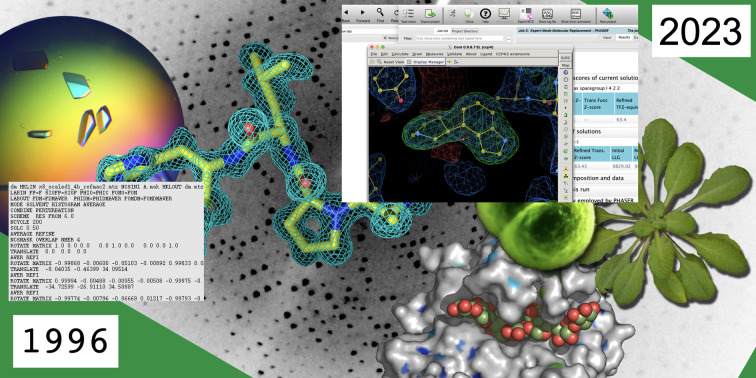
The evolution of CCP4 from 1996 to the present day.
